# Clinical benefit of MAO-B and COMT inhibition in Parkinson’s disease: practical considerations

**DOI:** 10.1007/s00702-023-02623-8

**Published:** 2023-03-24

**Authors:** Martin Regensburger, Chi Wang Ip, Zacharias Kohl, Christoph Schrader, Peter P. Urban, Jan Kassubek, Wolfgang H. Jost

**Affiliations:** 1 Department of Molecular Neurology, Friedrich-Alexander-Universität Erlangen-Nürnberg, Erlangen, Germany; 2 Department of Neurology, University Hospital of Würzburg, Würzburg, Germany; 3 Department of Neurology, University of Regensburg, Regensburg, Germany; 4 Department of Neurology, Hannover Medical School, Hannover, Germany; 5 Abt. für Neurologie, Asklepios Klinik Barmbek, Hamburg, Germany; 6 Department of Neurology, University Hospital Ulm, Ulm, Germany; 7 Parkinson-Klinik Ortenau, Wolfach, Germany

**Keywords:** Parkinson’s disease, MAO-B inhibitors, COMT inhibitors, Motor fluctuations

## Abstract

Inhibitors of monoamine oxidase B (MAO-B) and catechol-O-methyltransferase (COMT) are major strategies to reduce levodopa degradation and thus to increase and prolong its effect in striatal dopaminergic neurotransmission in Parkinson’s disease patients. While selegiline/rasagiline and tolcapone/entacapone have been available on the market for more than one decade, safinamide and opicapone have been approved in 2015 and 2016, respectively. Meanwhile, comprehensive data from several post-authorization studies have described the use and specific characteristics of the individual substances in clinical practice under real-life conditions. Here, we summarize current knowledge on both medication classes, with a focus on the added clinical value in Parkinson’s disease. Furthermore, we outline practical considerations in the treatment of motor fluctuations and provide an outlook on ongoing studies with MAO-B and COMT inhibitors.

## Motor fluctuations in Parkinson’s disease

Parkinson’s disease (PD) is the second most common neurodegenerative disorder, coined by progressive motor and non-motor symptoms affecting multiple aspects of health-related quality of life (Kuhlman et al. 2019). Worldwide prevalence has increased in recent decades, in part due to increasing life expectancy (Dorsey et al. 2018). During the initial phase of the disease, symptoms are usually well controlled with levodopa or dopamine agonists, but are particularly complicated by motor fluctuations later on.

### Risk factors for motor fluctuations

Motor fluctuations are inevitable during the course of PD treatment. Emerging motor fluctuations were observed after only 9 months in 20% of levodopa treated PD patients of the ELLDOPA trial (Fahn et al. 2004) and in 75% after 3 years of follow-up in the STRIDE-PD trial (Olanow et al. 2013). Earlier onset of motor fluctuations has been associated with younger age at onset, higher levodopa doses, and lower body weight (Olanow et al. 2013). Eventually, nearly all PD patients developed motor fluctuations in long-term studies (Ahlskog and Muenter 2001; Hely et al. 2005). Still, motor fluctuations were not provoked upon early start of low doses of levodopa in the LEAP trial (Verschuur et al. 2019). Similar conclusions were drawn from a 14-year follow-up of the PDRG-UK trial, comparing long-term outcomes after initial start of levodopa vs. levodopa/selegiline vs. bromocriptine (Katzenschlager et al. 2008). In this study, levodopa yielded superior results regarding health-related quality of life, without altered prevalence in motor fluctuations. In addition, the comparison of early initiation of levodopa in Italian versus late initiation in sub-Saharan African PD patients showed that motor fluctuations rather depend on disease duration as compared to cumulative levodopa exposure (Cilia et al. 2014). Finally, continuous dopaminergic stimulation via intestinal application of levodopa/carbidopa gel reduces motor fluctuations despite higher amounts of total doses applied (Antonini et al. 2016). Taken together, these studies led to the current concept that motor fluctuations in PD are primarily related to disease duration (i.e., neurodegeneration-associated loss of presynaptic storage and imbalance with other neurotransmitters) together with phasic dopaminergic stimulation in its pharmacotherapy (i.e., low half-lives of drugs, delayed gastric emptying and intestinal transport, impaired intestinal absorption) (Armstrong and Okun 2020). In conclusion, early administration of excess doses of levodopa should be avoided, and to this end, early administration of dopamine agonists, MAO-B inhibitors, and amantadine, alone or in combination is a valid strategy. However, there are conflicting goals between efficient symptom relief, tolerability, and short-/long-term side effects. Hence, current treatment guidelines recommend to outweigh patient-specific factors and preferences when choosing between levodopa as initial therapy or levodopa-sparing regimen (Lang and Lees 2002; Goetz et al. 2005; Horstink et al. 2006; Fox et al. 2018; Pringsheim et al. 2021).

### Classification of motor fluctuations

Although disease modification might be regarded as the major challenge, the practical treatment of motor fluctuations remains one of the major unmet needs in PD, evidenced by the number of new substances that are currently in clinical development for motor fluctuations in PD (McFarthing et al. 2022).

Levodopa-related motor fluctuations in PD comprise—

- *wearing-off*, i.e., progressive shortening of the time of benefit from levodopa < 4 h,
- *delayed time to ON*, i.e., delayed benefit after levodopa dosing,
- *on–off phenomena*, i.e., rapid fluctuations between ON and OFF states,
- *no-on*, i.e., lack of a meaningful benefit from a single levodopa dose, and
- *levodopa-induced peak-dose dyskinesia* or *diphasic dyskinesia* (Armstrong and Okun 2020).

The burden of OFF-episode-related disability is further increased by associated non-motor phenomena, such as pain derived from rigidity and dystonia, anxiety, depression, and fatigue (Storch et al. 2013; Martínez‐Fernández et al. 2016). Moreover, PD patients tend to prefer dyskinetic states over hypokinetic/OFF states (Hung et al. 2010). To this end, the reduction of daily OFF time is a meaningful goal in the treatment of middle and advanced stage PD and has been chosen as the primary endpoint in the majority of clinical trials with MAO and COMT inhibitors.

Quantification of motor fluctuations For clinical trials, the Hauser diary has been widely used to quantify time with motor fluctuations at home, but may underestimate ON time with dyskinesia (Hauser et al. 2000; Löhle et al. 2022). The initial version of the Wearing-Off Questionnaire consisted of 32 items and was designed for application in study settings (Stacy et al. 2005). Its simplified nine-item version is also suitable for application in daily routine (Stacy 2010). Part IV of the Unified Parkinson’s Disease Rating Scale (MDS-UPDRS) and the Unified Dyskinesia Rating Scale (UDysRS) are rater-applied scales to inquire or to directly examine OFF and ON symptoms. Recently, sensor-based systems for remote monitoring of motor fluctuations in PD, such as the Parkinson’s KinetiGraph (Woodrow et al. 2020), the Mobile GaitLab (Jakob et al. 2021), and the McRoberts MoveMonitor (Mikolaizak et al. 2022), have been developed, but have not found their way into therapeutic decision-making in the medical care setting yet. Hence, in clinical practice, the classical way of identifying motor fluctuations is history-based (Box 1).

**Box 1:** Exemplary questions to probe for motor fluctuations in PD patients (Jost et al. 2022).

- Have you noticed that the effect of your medication does not last until the next dosing?
- Have you experienced slowness or muscle cramps in the morning?
- Have you noticed tremor, stiffness, reduced dexterity, attacks of fear or panic, changes in mood, abnormal sensations or pain?
- Do these symptoms usually improve after your next dose?

### Rationale of inhibition of levodopa degradation in motor fluctuations

When confronted with motor fluctuations in PD, different strategies may be employed to flatten fluctuations of striatal dopaminergic stimulation. Before invasive therapeutic options are considered, this may be achieved by adaptions of oral therapy, i.e., dopamine agonists, increased fractionation of levodopa doses, use of prolonged formulations of levodopa as well as inhibition of levodopa degradation by monoamine oxidase B (MAO-B) inhibitors and catechol-O-methyltransferase (COMT) inhibitors. As new substances were recently approved for the latter classes, the present review article summarizes existing knowledge on MAO-B and COMT inhibitors, with a focus on existing knowledge from pivotal trials as well as post-approval evidence. Dopamine may be degraded by both MAO and COMT (Webster 2001). The production of the final product homovanillic acid requires both MAO and COMT.

## MAO-B inhibitors

Monoamine oxidase B (MAO-B) is an enzyme located at the outer mitochondrial membrane which catalyzes the oxidation of arylalkylamine neurotransmitters, and is the main degradation pathway of dopamine into 3,4-dihydroxyphenylacetic acid within striatal glial cells (Binda et al. 2004). Three MAO-B inhibitors have been approved: selegiline, rasagiline, and safinamide.

### Selegiline

Selegiline is a selective inhibitor of MAO-B and was studied in PD as early as in the 1970’s (Riederer et al. 1978). Different studies have demonstrated symptomatic benefit with low to moderate effects. For monotherapy of early PD, the randomized placebo-controlled DATATOP trial showed a delay in the clinical need for levodopa initiation (Parkinson Study Group 1996), and a benefit was still present in a double-blind continuation when adding levodopa (Shoulson et al. 2002). A more recent study confirmed improved motor scores upon selegiline treatment vs. placebo (– 6.3 vs. – 3.1 points of the sum score of UPDRS-I, -II, and -III) after 12 weeks (Mizuno et al. 2017). Earlier indications for a potential neuroprotective effect of selegiline remained inconsistent (Tábi et al. 2020).

Due to the irreversible inhibition of MAO-B and the slow turnover of cerebral MAO-B, enzyme function recovers only weeks after its discontinuation as indicated by a PET study in few subjects (Fowler et al. 1994). However, as MAO-B has to be inhibited by at least 80% to affect striatal dopamine levels, the net clinical effect of irreversible MAO-B inhibitors amounts to less than 14 days (Green et al. 1977; EMA 2019a). Selegiline is metabolized via methamphetamine to amphetamine which is not clinically relevant at the approved doses of 5 and 10 mg, but may lead to false-positive amphetamine derivate drug screening results. Interestingly, added clinical benefit from 10 mg vs. 5 mg is controversial (LeWitt 2009). At higher doses, selectivity for MAO-B decreases. Its use is contraindicated in combination with sympathomimetic drugs, serotonergic drugs (including opioids, pethidine, serotonin reuptake inhibitors, and serotonin-norepinephrine reuptake inhibitors), tricyclic antidepressants, other MAO inhibitors, and serotonin antagonists including triptans (Csoti et al. 2012). In combination with levodopa, multiple contraindications have to be considered according to the European label, which has largely limited the clinical use of selegiline (gastric or duodenal ulcers, arterial hypertension, hyperthyroidism, pheochromocytoma, glaucoma, prostate hyperplasia with urinary retention, tachycardia, cardiac arrhythmia, severe coronary heart disease, psychiatric disorders, and dementia).

### Rasagiline

Like selegiline, rasagiline causes a selective and irreversible inhibition of MAO-B, although at 5–10 times lower doses (Finberg et al. 1996). A complete inhibition of MAO-B is achieved after few days of therapy, but clinical response may take up to 4 weeks in some individuals. Due to the irreversibility of inhibition, the half-life of its effect also depends on de novo synthesis of MAO-B (Freedman et al. 2005). Rasagiline is mainly metabolized into aminoindane, and the potential of serotonergic interactions is considered less problematic (Smith et al. 2015). Contraindications include combination with other MAO inhibitors and with pethidine, and it should be administered carefully in combination with other serotonergic compounds (EMA 2019a).

Similarly to selegiline, a modest symptomatic effect of rasagiline has been demonstrated when initiated as monotherapy in early PD. In the TEMPO study, significant benefits of rasagiline 1 mg and 2 mg were shown in comparison to placebo on total UPDRS scores (mean difference to placebo after 24 weeks – 4.2 for 1 mg; – 3.6 for 2 mg) as well as on its motor and ADL subscales (Parkinson Study Group 2002). Different studies with early vs. delayed start study design indicated a potential disease modifying effect of rasagiline which was, however, not confirmed in the long-term follow-up (Parkinson Study Group 2004; Olanow et al. 2009; Rascol et al. 2011, 2016). In PD patients with levodopa-induced motor fluctuations, two trials showed clear benefit of rasagiline: first, in the LARGO trial, 1 mg rasagiline yielded increased daily ON time (+ 0.85 h compared to placebo) without increasing time with troublesome dyskinesia, improved CGI and UPDRS part IV, and was non-inferior to entacapone (Rascol et al. 2005). In the PRESTO trial, 1 mg rasagiline decreased daily OFF time (– 0.9 h compared to placebo) and increased daily ON time and ON time with troublesome dyskinesia (Parkinson Study Group 2005).

### Safinamide

Unlike selegiline and rasagiline, the inhibition of MAO-B by safinamide is reversible, i.e., its functional half-life is not dependent on the neosynthesis of MAO-B and therefore mainly depends on the decay of safinamide, with a half-life of 22 h. Moreover, safinamide is an α-aminoamide and has additional pharmacological effects in terms of a modulation of excessive glutamate release and modulation of voltage-dependent sodium channels although the relevance of these additional functions remains unclear (EMA 2019b). An initial open-label study of increasing doses of safinamide in PD showed added clinical benefits even when MAO-B inhibition was already achieved, but it remains debatable whether these functions are clinically relevant (Marzo et al. 2004; Stocchi et al. 2006). As for selegiline and rasagiline, there is no relevant interaction with tyramine ingestion (Cattaneo et al. 2003; Stefano and Rusca 2011; Marquet et al. 2012).

Positive effects of safinamide were demonstrated after 24 weeks in a total of four large randomized controlled trials, and after 2 years in one blinded extension study. In the “016” study, safinamide vs. placebo was assessed in addition to levodopa and optional additional dopaminergic therapy in mid- to late-stage motor fluctuations. Safinamide improved daily ON time compared to placebo (+ 0.4 h both for 50 mg and 100 mg safinamide), daily OFF time (– 0.4 h both for 50 mg and 100 mg safinamide), UPDRS part III (– 2.6 points for 50 mg, and – 1.8 for 100 mg safinamide), and CGI-C (Borgohain et al. 2014a). In the 18 months of extension study (“018”) maintaining blinding, the primary endpoint of change in Dyskinesia Rating Scale (DRS) total score was not significant, but showed maintained positive effects on daily ON time and daily OFF time (Borgohain et al. 2014b). As DRS scores were rather low in most participants at baseline, a post hoc analysis in participants with intermediate or severe dyskinesia at baseline showed a significant benefit of safinamide doses on DRS scores (Cattaneo et al. 2015). During the screening period of the SETTLE study, medication was optimized to minimize motor fluctuations, and only patients with persistent daily OFF time of at least 1.5 h underwent randomization. Safinamide (increased to 100 mg in 91% of patients after 2 weeks) increased daily ON time without troublesome dyskinesia by 1.0 h compared to placebo, and reduced daily OFF time by 1.0 h (Schapira et al. 2016). Significant improvements were also observed for UPDRS part III, for the proportion of patients with improvement on CGI-C, for PDQ-39 scores, CGI-C scores, PGI-C scores and CGI-S scores, for OFF time after the morning levodopa dose and for EQ-5D scores. The Japanese “ME2125-3 “ study confirmed significant benefits of safinamide vs. placebo after 24 weeks in PD patients with wearing-off on levodopa treatment, regarding daily ON time (+ 1.4 h at 50 mg, + 1.7 h at 100 mg) and additional measures including daily OFF time, and the UPDRS subscores for parts I, II, and III (Hattori et al. 2020). Recently, data of the Chinese phase III, double-blind, placebo-controlled, pivotal XINDI trial have been published which confirmed a positive effect of safinamide on motor fluctuations and motor symptoms (Wei et al. 2022).

In the European post-authorization observational SYNAPSES trial of 1558 patients, no new safety concerns evolved, especially in the subgroup of patients > 75 years of age (Abbruzzese et al. 2020). In the subgroup of patients with comorbidities, adverse events and serious adverse events were more frequent than in PD patients without additional relevant diseases, but did not show increased frequency for a single adverse event other than dyskinesia. No case of serotonergic syndrome was observed although 43% of participants had a psychiatric comorbidity, matching findings from a single-center retrospective analysis of 25 PD patients on dual safinamide and anti-depressive therapy (Pérez-Torre et al. 2021). Likewise, in an observational German study of 299 PD patients, no novel adverse events were recorded, and positive changes compared to baseline were observed for motor and non-motor outcomes (Jost et al. 2018).

## COMT inhibitors

COMT catalyzes the conversion of levodopa into 3-O-methyl-DOPA (3-OMD). COMT is expressed in all peripheral tissues and in the central nervous system (CNS) while the peripheral component is critical for the CNS bioavailability of levodopa. Peripheral COMT not only reduces levodopa concentrations, but its product 3-OMD also competes with levodopa in terms of blood–brain barrier transport and CNS dopamine uptake, and thus worsens motor function (Wade and Katzman 1975; Nyholm 2006; Adamiak et al. 2010; Adamiak-Giera et al. 2021). For this reason, COMT inhibition acts not only by increasing levels of levodopa transported into the CNS, but also through a sustained reduction of its competitor 3-OMD, an effect that is potentiated upon presence of dopamine decarboxylase inhibitors (DDCI) (Bonifacio et al. 2012). Three COMT inhibitors have been approved: tolcapone, entacapone, and opicapone.

### Tolcapone

In contrast to entacapone and opicapone, tolcapone crosses the blood–brain barrier due to its lipophilic structure, which leads to an inhibition of COMT both in the periphery and in the CNS (Ceravolo et al. 2002). In 1997, Tolcapone was the first COMT inhibitor to be approved for PD with motor fluctuations, after demonstrating significant reduction of OFF time in randomized placebo-controlled studies (Baas et al. 1997; Kurth et al. 1997; Adler et al. 1998; Rajput et al. 1998). There was a meaningful reduction of daily OFF time compared to placebo (100 mg vs. placebo: – 8.5% points of daily OFF time; 200 mg vs. placebo: – 5.6% points, corresponding to about – 0.85 and – 0.56 h of absolute OFF time reduction, respectively; Baas et al. 1997). Four cases of liver injury, three of them with a fatal outcome, led to the suspension of marketing in 1998. Hepatic toxicity may be derived from the depolarizing effect of tolcapone on mitochondria, whereas this effect was not present for entacapone (Nissinen et al. 1997; Gerlach et al. 2003). The suspension was lifted in 2004, along with narrowing of the label to cases that do not respond to or do not tolerate other COMT inhibitors, and with the obligation of regular liver function tests (Olanow and Watkins 2007; Artusi et al. 2021). This has substantially limited the practical role of tolcapone.

### Entacapone

As the second within the class of COMT inhibitors, entacapone was approved in 1998. It is administered at a fixed dose of 200 mg concomitant with each dose of levodopa and DDCI, due to its short half-life similar to levodopa. Similar to tolcapone, entacapone leads to less-pulsatile profiles of levodopa plasma levels (Nutt et al. 1994). A fixed combination of entacapone, levodopa, and carbidopa within one tablet eases dosing, while more complex dosing regimen may be required in select complicated cases (Brusa et al. 2004; Koller et al. 2005). Randomized, placebo-controlled studies of entacapone in PD patients with motor fluctuations showed an increase in ON time, a decrease in OFF time, a reduction of daily levodopa doses as well as improved UPDRS motor and ADL scores. The pivotal randomized placebo-controlled trials of entacapone were conducted on 205 and 171 patients, respectively (Parkinson Study Group 1997; Rinne et al. 1998). In the pivotal trial, a reduction of daily OFF time of 1.2 h when compared to placebo was observed (Rinne et al. 1998). Additional trials confirmed beneficial effects of entacapone, including the 3-year open-label extension part (Larsen et al. 2003) and placebo-controlled trials performed in the United Kingdom (Brooks et al. 2003), in Germany/Austria (Poewe et al. 2002), and in Italy when evaluating different timings of entacapone administration (Brusa et al. 2004). Positive effects of entacapone regarding global clinical impression and, less consistently, health-related quality of life were also inferred from the pivotal and open-label follow-up studies (Fénelon et al. 2003; Larsen et al. 2003; Onofrj et al. 2004; Koller et al. 2005; Reichmann et al. 2005).

### Opicapone

As the third COMT inhibitor, opicapone was approved in 2016. Like entacapone, it acts in the periphery only and does not affect activity of COMT in the brain, but it remains unknown if this adds to an increased tolerability (Kiss et al. 2010). As determined with rising oral doses of opicapone in healthy individuals, unbound opicapone has a low elimination half-life of about 1 h, but led to a prolonged inhibition of erythrocyte COMT, compatible with a strong association to the COMT enzyme that had previously been suggested by computer simulations (Palma et al. 2012; Almeida et al. 2013). Repeated doses of opicapone led to a long-lasting inhibition of > 100 h, allowing a once daily dosing regimen (Almeida et al. 2013; Rocha et al. 2013). Since unbound opicapone was eliminated with a half-life of 1.4 h, there was no accumulation of opicapone (Rocha et al. 2013). Restoration of COMT activity was dependent on the slow dissociation of the opicapone-COMT complex (Rocha et al. 2013).

Although concomitant food intake reduced opicapone resorption by about 20%, its functional effect on COMT inhibition was not significantly altered when taken with or without meals (Almeida et al. 2013). There were no obvious differences of the effect of opicapone on COMT inhibition comparing its intake in the morning and in the evening, but congruent with its application in the pivotal studies, the recommended intake is before bedtime with an interval of at least 1 h to levodopa dosing (Ferreira et al. 2015; EMA 2019c).

Bi-Park 1 was a randomized, double-blind trial of opicapone in PD with end-of-dose motor fluctuations and included 590 patients assigned to placebo, entacapone and opicapone at 5 mg, 25 mg and 50 mg, respectively (Ferreira et al. 2016). After 15 weeks of treatment, opicapone 50 mg was superior to placebo (– 1.0 h) and non-inferior to entacapone (– 0.2 h) regarding reduction of daily OFF times, and superior to placebo (+ 1.2 h) and non-inferior to entacapone (+ 0.3 h) for increase of daily ON times. Similar effects were shown for ON time without troublesome dyskinesia and clinician’s and patient’s global impression of change, while there were no significant differences for total UPDRS scores, PDQ-39 scores and the NMSS scores. In the double-blind Bi-Park 2 study, 427 patients were randomized to placebo, opicapone 25 mg and opicapone 50 mg (Lees et al. 2016). After 15 weeks, there was a significant – 0.9 h reduction of OFF time for the 50 mg dose compared to placebo, and the amount of OFF time reduction was maintained until the end of the subsequent 1 year open-label extension phase. Opicapone 50 mg was compared to placebo in a combined post-hoc analysis of both Bi-Park studies which allowed stratification for different subgroups of a total of 517 patients (Antonini et al. 2020; Rocha et al. 2021). This analysis indicated that the positive effects of opicapone on daily OFF time were more pronounced in “earlier” stages of motor fluctuations, characterized by lower Hoehn & Yahr stages, less and lower daily levodopa doses, and shorter disease duration. While these results may be caused by insufficient fractionation and adaptation of levodopa and dopamine agonists to control motor fluctuations in this “early” population, they still provide evidence that opicapone is effective at different stages of motor fluctuations, including patients that do not receive dopamine agonists or MAO-B inhibitors. In the Japanese pivotal COMFORT-PD trial, 437 PD patients were randomized to placebo, opicapone 25 mg or opicapone 50 mg, and a significant reduction of daily OFF time (– 0.7 and – 0.6 h, respectively, compared to placebo) was observed for both doses after 15 weeks of treatment (Takeda et al. 2021).

While the open-label design of subsequent studies only provides indirect indications for efficacy, their results still deliver valuable information for the use of opicapone in real-world settings and in a broader population of patients. In the post-approval open-label OPTIPARK study, 393 PD patients with motor fluctuations who were prospectively followed up for at least 3 months after initiation of opicapone 50 mg showed a significant improvement of the UPDRS ADL subscore and the UPDRS motor subscore in the ON condition (Reichmann et al. 2020). There were also significant improvements of the PDQ-8 and the NMSS scales.

**Table 1:** Overview of the main characteristics of MAO-B and COMT inhibitors in PD

Substance	Label (EMA)	Dosing	Approx. half-life of enzymatic inhibition	Main side effects	Caveats
*MAO-B inhibitors*					
Selegiline	Monotherapy, or combination with levodopa/DDCI	5 mg or 10 mg OD in the morning	14 days	Insomnia, bradycardia, hypotension, nausea	Multiple interactions and contraindications (see text)
Rasagiline	Monotherapy, or combination with levodopa/DDCI in end-of-dose fluctuations	1 mg OD in the morning	14 days	Nausea, light-headedness, headache, insomnia	Contraindication in combination with other MAO inhibitors and pethidine; increased caution in combination with other serotonergic compounds
Safinamide	Combination with a stable dose of levodopa/DDCI ± other PD drugs in mid-to late-stage fluctuating patients	50 mg OD in the morning, increase to 100 mg OD after 2 weeks as needed	22 h	Dyskinesia, hallucinations, nausea	Contraindication in combination with other MAO inhibitors and pethidine, severe hepatic impairment, certain eye diseases. Increased caution in combination with other serotonergic compounds
*COMT inhibitors*					
Tolcapone	Idiopathic PD with motor fluctuations, non-responsive to or intolerant of other COMT inhibitors in combination with levodopa/DDCI	100 mg TID or 200 mg TID	3–4 h	Diarrhea, urine discoloration, dyskinesia	Third line COMT substance. Strict vigilance for liver injury by liver function tests every 2 weeks for 1 year, every 4 weeks for subsequent 6 months and every 8 weeks thereafter
Entacapone	PD with end-of-dose motor fluctuations in combination with levodopa/DDCI	200 mg with each levodopa/DDCI dose	2–3 h	Diarrhea, urine discoloration, dyskinesia	Contraindication in combination with *non-selective* MAO inhibitors, in hepatic impairment and previous neuroleptic malignant syndrome
Opicapone	Combination with levodopa/DDCI in end-of-dose fluctuations	50 mg OD in the evening	4 days	Dyskinesia	Contraindication in combination with MAO inhibitors other than those used for PD, previous neuroleptic malignant syndrome

Information presented in abbreviated form from the respective European Public Assessment Report Product Information

*EMA* European Medicines Agency, *MAO-B* monoamine oxidase B, *COMT* catechol-O-methyltransferase, *DDCI* DOPA decarboxylase inhibitor, *OD* once daily, *TID* trice daily

## Practical considerations

### When a patient develops wearing-off, is the addition of a MAO-B / COMT inhibitor superior to adjusting levodopa dosing?

In PD patients starting to experience wearing-off phenomena, different types of dopaminergic therapy adaptation come into consideration. Increased numbers of doses and/or decreased intervals of levodopa/DDCI application may provide benefit, but are associated with an increased burden for patients and may lead to even more pulsatile levels of striatal levodopa.

Starting rasagiline is an in-label option to counteract emerging wearing-off phenomena with a similar effect as entacapone, as demonstrated by high-class evidence in LARGO and in PRESTO (Parkinson Study Group 2005; Rascol et al. 2005). Safinamide has been approved for mid- to late-stage fluctuating PD patients only, because the pivotal study required a minimum of 1.5 h of daily OFF time after adjusting levodopa dosing (Borgohain et al. 2014a; Schapira et al. 2016). However, no high-class evidence exists for early-stage fluctuations to date, but may well be effective at these stages. For entacapone and opicapone, the European label requires that end-of-dose motor fluctuations cannot be stabilized by levodopa, but it is up to the treating clinician how many adaptations of the levodopa regimen are deemed necessary or justifiable for the individual patient. Extended release formulations of levodopa are chosen to improve OFF periods at night and in early mornings, and a post hoc analysis of the Bi-Park study cohort showed that opicapone 50 mg significantly reduced early morning OFF periods (Videnovic et al. 2020). In a phase 1 study, opicapone increased overall levodopa exposure also in individuals who were on monotherapy with extended release formulations of levodopa (Loewen et al. 2021). While a head-to-head comparison of the effects of levodopa extended release and opicapone on sleep in PD is missing, the OPTIPARK open-label study showed an improvement of sleep upon initiation of opicapone (Reichmann et al. 2020).

Existing randomized trials of COMT inhibitors in early PD without motor fluctuations have yielded equivocal results potentially related to study designs, and the results of the ongoing ADOPTION and EPSILON studies will provide more insights on the benefits of earlier time points to initiate therapy with COMT inhibitors (see Sect. Open questions and ongoing studies on MAO-B and COMT inhibitors in PD).

### Which scenarios justify a within-class change of the MAO-B or COMT inhibitor?

In fact, due to its label also for treatment early in the PD course, development of wearing-off in patients who had been on rasagiline for a long time will pose the question whether a switch from rasagiline to safinamide may lead to a better control of motor fluctuations. No head-to-head studies of higher evidence exist comparing rasagiline and safinamide, but the additional anti-glutamatergic mechanism provides a theoretical basis for additional benefit. In a small retrospective open-label series of 17 subjects with fluctuations while under levodopa and rasagiline, changing to safinamide 100 mg resulted in a benefit in nine patients (Bianchini et al. 2021). According to the product information, an interval of at least 14 days must elapse before starting a different MAO-B inhibitor after stopping rasagiline (EMA 2019a). After stopping safinamide, an interval of 7 days is demanded (EMA 2019b). Nevertheless, a seamless switch from rasagiline to safinamide 50 mg may be considered in practice, in order to circumvent a clinical worsening of dyskinesias in a situation when a therapeutic change is required. In an earlier case series of 30 PD patients, an overnight switch from selegiline to rasagiline produced no side effects and no increase in blood levels of amphetamine derivates (Müller et al. 2013). In a recent study on 20 PD patients on rasagiline including four patients with concomitant SSRI medication, an overnight switch to safinamide 50 mg and subsequent increase to 100 mg after 14 days did not produce changes in concomitant ECG and blood pressure recordings (Stocchi et al. 2021). In any way, change of therapy requires thorough education of patients about potential clinical signs of exceptional side effects such as serotonergic syndrome and common side effects like increase of dyskinesias which necessitates subsequent adaptation of levodopa dosing. To this end, a clinical follow-up 2–4 weeks after changes in MAO-B or COMT inhibitors seems plausible in many cases while in advanced stage patients, medication changes are preferably implemented in an inpatient setting.

The simplified dosing regimen as well as the lack of gastrointestinal side effects and urine discoloration justify a switch from tolcapone or entacapone to opicapone when these symptoms cause problems, especially in patients with impaired gastrointestinal motility which also impairs resorption of tolcapone or entacapone.

In addition to these advantages, an additional added clinical benefit of opicapone over entacapone has been shown indirectly. In the Bi-Park 1 study, there were no significant changes of opicapone 50 mg over entacapone during the double-blind study phase in the primary and secondary outcomes, but it was only powered to show non-inferiority of both treatment arms (Ferreira et al. 2016). Due to their short half-lives, switching from tolcapone or entacapone to opicapone can be performed without concern; in the Bi-Park 1 open-label extension study, the double-blind entacapone arm started open-label opicapone in the evening of the last day of entacapone therapy which resulted in a significant reduction in daily OFF times by 0.6 h until the end of the study (post hoc and not adjusted for multiplicity of testing) (Ferreira et al. 2018).

According to a recent network-based meta-analysis, tolcapone had highest efficiency regarding ON time, UPDRS part III scores and reduction of levodopa daily dose equivalent. Taking safety data into account, however, opicapone was determined superior to both entacapone and tolcapone (Song et al. 2021).

### Does rasagiline have an added benefit in later stages of Parkinson’s disease?

Due to good tolerability and the once daily formulation, rasagiline is frequently chosen as initial therapy in early stages of PD when a modest symptomatic effect (– 4.2 points of total UPDRS scores vs. placebo) is expected to be sufficient (Parkinson Study Group 2002). In PD with motor fluctuations, both the LARGO and PRESTO study documented significant positive effects on daily OFF and ON times, albeit at lower absolute ranges compared to the pivotal trials of safinamide, entacapone and opicapone. Different study protocols and the lack of head-to-head clinical trials, however, preclude a direct comparison.

### Which patients benefit from a combined therapy with both MAO-B and COMT inhibitors?

Concomitant use of MAO-B inhibitors was present in about 20% of each treatment arm in the Bi-Park 1 trial for opicapone (Ferreira et al. 2016), and concomitant use of rasagiline or selegiline with opicapone is permissible according to the label, whereas caution was advised with safinamide due to a lack of data (EMA 2019c). In the post-approval open-label study OPTIPARK for opicapone, comedication with rasagiline was present in 136 individuals and with safinamide in 67 individuals (Reichmann et al. 2020). These subgroups were not specifically addressed in this report, but treatment emergent adverse events corresponded to data from the pivotal trials, providing first indications that combination with safinamide is safe also under real-world conditions. There are no data, however, on the question whether combined application of MAO-B and COMT inhibitors provide a specific benefit in certain populations of PD patients.

### In patients treated with jejunal levodopa infusion, are there positive effects of the addition of MAO-B and COMT inhibitors?

Continuous intestinal infusions of levodopa/carbidopa (LCIG) or levodopa/carbidopa/entacapone (LECIG) are approved invasive therapeutic options for the treatment of motor fluctuations that cannot be adequately controlled by non-invasive drug therapy. Although the actual goal of intestinal infusion therapy is the discontinuation of other dopaminergic drugs, the labels of both infusion combinations specifically allow concomitant use of MAO-B inhibitors, as well as COMT inhibitors for levodopa/carbidopa. In PD patients with high levels of intestinal levodopa infusion rates, addition of MAO-B or COMT inhibitors may help avoid the need of multiple changes of infusion cassettes per day. Moreover, pyridoxine levels are frequently reduced when levels of daily levodopa administration are high (Loens et al. 2017)**.** Two studies indicated that addition of an oral COMT inhibitor allows for a reduction of LCIG infusion rates. First, in a short-term pharmacokinetic study of 9 PD patients under LCIG, levodopa plasma levels remained stable when entacapone was introduced, and LCIG infusion rates were decreased by 20%; levodopa plasma levels even increased upon introduction of oral tolcapone and concomitant reduction of LCIG by 20% (Nyholm et al. 2012). The strong effect of tolcapone was further supported by a case series of four PD patients on LCIG where addition of tolcapone allowed for a relative dose reduction of LCIG of 19–50%, but all patients experienced hyperkinetic side effects during the dose adjustment period, and delirium was present in two patients (Schröter et al. 2020). Furthermore, a switch from LCIG to the recently approved LECIG therapy is an option to reduce daily levodopa doses; in a report on 12 patients switched directly from LCIG to LECIG, infusion rates of levodopa were reduced by a mean of 32.5% (Öthman et al. 2021). No reports were identified on the concomitant use of MAO-B inhibitors and LCIG. In summary, in PD patients treated with LCIG, addition of a COMT inhibitor (oral or LECIG) may be considered if a reduction of levodopa consumption is needed, but these patients require close monitoring in an inpatient setting during the dose adjustment period.

### Discontinuation of MAO-B and COMT inhibitors

As outlined in Table 1, the recovery of MAO-B and COMT enzyme activities differs substantially between the different substances, and has to be considered when pausing medication. In the circumstances of dose adjustments when a formal levodopa challenge test is to be performed, pausing of safinamide for few days should lead to sufficient decrease of its effects, whereas discontinuation of selegiline or rasagiline seems impracticable due to the decrease of enzymatic function for over 2 weeks, and due to their mild symptomatic effect in absence of levodopa. The effects of tolcapone and entacapone on COMT inhibition will diminish within few hours after discontinuation, whereas COMT inhibition of opicapone will persist for few days. This is why pausing of opicapone for a levodopa challenge test is not necessary since its action is mainly dependent on the presence of levodopa (Saranza and Lang 2021).

### How to manage psychosis in patients treated with MAO-B and/or COMT inhibitors?

In the setting of psychosis in PD, reduction of anti-Parkinson medication is often considered depending on its course and underlying cause. If reduction of dopaminergic medication is necessary, the following order has been proposed, with decreasing pro-psychotic potential: anticholinergics, amantadine, MAO-B-inhibitors, dopamine agonists, COMT inhibitors, levodopa extended release, levodopa (Seppi et al. 2011; Connolly and Lang 2014; Levin et al. 2016). Of note, psychosis was a rare adverse event upon MAO-B and COMT inhibitors in the pivotal trials (Parkinson Study Group 1996, 2002; Baas et al. 1997; Rinne et al. 1998; Borgohain et al. 2014a; Ferreira et al. 2016).

### Are there specific effects of MAO-B and COMT inhibition on non-motor symptoms?

Effects on non-motor symptoms have been analyzed for several of the MAO-B and COMT inhibitors. In a German observational study, initiation of rasagiline (or switch from selegiline) showed positive effects not only on daily OFF time, but also on aspects of quality of life as measured with the PDQ-39 scale (Jost et al. 2008). In a randomized, placebo-controlled study in PD patients with established dopaminergic therapy and mild cognitive impairment, rasagiline led to an improvement of cognitive measures which could, however, not be reproduced in another trial (Hanagasi et al. 2011; Weintraub et al. 2016).

The additional modulation of glutamate receptors and sodium channel inhibition is specific for safinamide within the MAO-B inhibitor class and might offer an appealing explanation for its effects on non-motor symptoms. In a prospective cohort of 13 PD patients with pain, open-label initiation of safinamide led to significant improvements of pain rating scales (KPSS, BPI, NRS) as well as motor scales (UPDRS parts III and IV, CGI-S, PDQ-39), but did not alter laser-evoked potentials as a surrogate marker of central processing of nociceptive inputs (Geroin et al. 2020). An open-label multi-center study demonstrated positive effects of safinamide in 27 PD patients on specific items of the King’s PD pain scale, on total scores of the King’s PD pain scale, and on UPDRS part IV, while unchanged values were reported for non-motor symptom scale, HADS, PDQ-8, PDSS-2, EuroQol-5D, CGI-I, and PGI-C (Grigoriou et al. 2021). Additional indirect evidence of positive effects of safinamide on pain in PD stem from a post hoc analysis of the combined “016” and SETTLE study, as well as the “018” extension study, demonstrating less frequency of concomitant therapy with analgesics in the safinamide group compared to placebo (Cattaneo et al. 2016, 2018). The Spanish descriptive, observational, longitudinal, prospective open-label study SAFINONMOTOR recruited 50 PD patients with an NMSS ≥ 40, and showed improvement of mood-related items on the NMSS and the PDQ-39 as well as sleep-related scores of the PSQI and the Epworth Sleepiness Scale (Labandeira et al. 2021; García et al. 2022a). Finally, in a randomized, longitudinal, cross-over study of 30 PD patients with REM sleep behavioral disorder in PD, improvement in sleep was reported in a subset of patients (Plastino et al. 2021). In the SURINPARK study, change of the scale for outcomes in Parkinson’s disease for autonomic symptoms in urinary symptoms (SCOPA-AUT-U) was improved upon safinamide, as determined retrospectively comparing 32 PD patients newly started on safinamide and 78 patients not treated with safinamide (Gómez-López et al. 2021).

Motor fluctuations, however, are inevitably connected with non-motor complaints (Martínez‐Fernández et al. 2016). This means that the presence of specific glutamatergic and sodium channel effects remains speculative. Moreover, the safinamide-mediated inhibition of glutamate release has not been demonstrated in humans. These data stem from in vitro data as well as ex vivo murine models (Salvati et al. 1999; Caccia et al. 2006). In vivo measurements of intracerebral glutamate release in safinamide-treated rats indicated that relevant effects might only be present at concentrations equivalent to a daily dose of at least 100 mg in humans (Morari et al. 2017). Nevertheless, in light of the proven effects on non-motor symptoms of safinamide, their precise mechanism might not be relevant for daily practice.

Previous trials suggested an improvement of specific non-motor symptoms also for COMT inhibitors. An open-label study of tolcapone showed a significant reduction of multiple items of the non-motor symptoms assessment scale for PD (NMSS) four weeks after baseline (Müller and TANIMOS Study Investigators 2014). Likewise, in a post hoc analysis of the BIPARK-II open-label phase, an improvement of multiple items of the NMSS was observed (Fabbri et al. 2018). Moreover, NMSS scores were reduced in the OPTIPARK open-label study of opicapone (Reichmann et al. 2020). In the open-label OPEN-PD trial conducted in Spain, 30 PD patients showed a reduction of NMSS scores by 27% after 6 months of follow-up (García et al. 2022b).

## Open questions and ongoing studies on MAO-B and COMT inhibitors in PD

While the use of MAO-B and COMT inhibitors is now well established in PD with motor fluctuations, ongoing studies aim to characterize their effects on non-motor symptoms in more detail and their use before the onset of motor fluctuations.

In the phase 4 double-blind randomized OCEAN trial, PD patients with wearing-off and PD associated pain will be treated with opicapone or placebo, in addition to their established levodopa treatment (Chaudhuri et al. 2022). The primary outcome is defined as change from baseline in the fluctuation-related pain domain of the King’s Parkinson’s Disease Pain Scale (KPSS). While this measure may well be indirectly attributable to an improvement of motor fluctuations, it is still of clinical relevance.

In the phase 4 open-label OASIS trial, the effect of opicapone added to a stable treatment with levodopa in PD patients with wearing-off and sleep problems will be evaluated, with a primary endpoint of the change from baseline in the total score of the Parkinson’s Disease Sleep Scale 2 (PDSS-2) (Costa et al. 2021).

With the availability of the latest generation of MAO-B and COMT inhibitors, their pharmacokinetics of a once daily application and their tolerability pose the question whether they can be used at earlier timepoints, before motor fluctuations emerge (Jenner et al. 2021). In this regard, the randomized, double-blind, placebo-controlled phase 3 EPSILON study aims to assess the effect of opicapone administered in PD patients without fluctuations, with a primary outcome measure of change in the UPDRS-III motor score (Ferreira et al. 2022). Previous studies on the early initiation of the classical COMT inhibitors in PD patients without motor fluctuations have remained equivocal. Early initiation of entacapone in addition to levodopa in the STRIDE-PD study did not result in a delayed onset of motor fluctuations when compared to levodopa alone, but might have been confounded by high doses of entacapone and levodopa (Stocchi et al. 2010). Additional studies showed that entacapone started in early PD patients led to more stable levodopa doses over the subsequent follow-up period, but this effect was lost upon discontinuation of entacapone (Poewe et al. 2002; Brooks et al. 2003; Hauser et al. 2009). Tolcapone started early in PD led to a smaller ratio of patients with motor fluctuations after 12 months (Waters et al. 1997).

In the ADOPTION study, initiation of opicapone directly upon the emergence of early motor fluctuations will be tested versus placebo (Ferreira et al. 2021).

It is established that high doses of oral levodopa favor the occurrence of motor fluctuations in the long-term (Tran et al. 2018), but it remains speculative whether this is facilitated by altered plasticity within the basal ganglia due to persistently changing CNS levodopa concentrations. To this end, continuous dopaminergic stimulation by levodopa may be a mechanism to delay motor fluctuations (Picconi et al. 2012; Jost 2022).

## Concluding remarks

Inhibitors of MAO-B and COMT have become a highly valuable component in the therapy of PD patients when motor fluctuations cannot be adequately controlled by adjusting levodopa dosing. Reduction of OFF time, increase in ON time as well as reduction of daily levodopa doses have been demonstrated for all substances, and additionally, positive effects on non-motor symptoms are emerging. Safinamide and opicapone currently represent the latest generations of both classes, and their efficacy and safety profiles have now been well characterized in the pivotal studies as well as a variety of subsequent phase 4 studies.

## Data Availability

Data sharing not applicable to this article as no datasets were generated or analysed during the current study.
